# Prevalence and risk factors of airflow limitation in a Mongolian population in Ulaanbaatar: Cross-sectional studies

**DOI:** 10.1371/journal.pone.0175557

**Published:** 2017-04-11

**Authors:** Motoyuki Nakao, Keiko Yamauchi, Yoko Ishihara, Hisamitsu Omori, Bandi Solongo, Dashtseren Ichinnorov

**Affiliations:** 1 Department of Public Health, School of Medicine, Kurume University, Kurume, Fukuoka, Japan; 2 Department of Biomedical Laboratory Sciences, Faculty of Life Sciences, Kumamoto University, Kumamoto, Japan; 3 Department of Respiratory Medicine, Mongolian National University of Medical Sciences, Ulaanbaatar, Mongolia; Liverpool School of Tropical Medicine, UNITED KINGDOM

## Abstract

The burden of chronic obstructive pulmonary disease (COPD) is expected to increase in the coming decades. In Ulaanbaatar, Mongolia, air pollution, which has been suggested to correlate with COPD, is a growing concern. However, the COPD prevalence in Ulaanbaatar is currently unknown. This study aims to estimate the prevalence of airflow limitation and investigate the association between airflow limitation and putative risk factors in the Mongolian population. Five cross-sectional studies were carried out in Ulaanbaatar. Administration of a self-completed questionnaire, body measurements, and medical examination including spirometry were performed in 746 subjects aged 40 to 79 years living in Ulaanbaatar. The age- and sex-standardized prevalence of airflow limitation in Ulaanbaatar varied widely from 4.0 to 10.9% depending on the criteria for asthma. Age, body mass index (BMI), and smoking habit were independent predictors for airflow limitation while residential area and household fuel type were not significant. In conclusion, prevalence of putative COPD was 10.0% when subjects with physician-diagnosed asthma were excluded from COPD. Older age, lower BMI, and current smoking status were putative risk factors for airflow limitation. This prevalence was consistent with reports from Asian countries.

## Introduction

Chronic obstructive pulmonary disease (COPD) is characterized by persistent airflow limitation caused by a mixture of small airway disease and parenchymal destruction [1]. According to WHO estimates, COPD is the third leading cause of death globally [2]. There were 3.1 million COPD deaths in 2012, corresponding to 5% of all deaths worldwide. The morbidity and mortality of COPD, and its associated economic and social burdens, are increasing globally even in the low- and middle-income countries [3, 4]. The most evident risk factor for COPD is cigarette smoking, with COPD prevalence correlating with tobacco smoking prevalence. In developing countries, outdoor and indoor air pollution is also a major risk factor of COPD [5]. The prevalence and burden of COPD are expected to increase in the coming decades due to high smoking rates and severe air pollution in developing countries, and population aging in developed countries.

COPD prevalence varies according to survey methods, diagnostic criteria, and analytical methods. The prevalence of physiologically defined COPD in adult subjects over 40 years has been reported to be 9–10% although there are regional or methodological differences [6]. Amongst Asian countries, COPD prevalence has been reported to be 10.9% in Japan [7], 13.7% in Korea (data from the Korea National Health and Nutrition Examination Survey) [8], 8.2% and 11.4% in China [9], and 13.9% in Manila, Philippines [10].

Ulaanbaatar, the Mongolian capital, is one of the world's worst air-polluted cities and concerns about air pollution are growing [11]. Coal and biomass fuel consumption is the major cause of indoor and outdoor air pollution in Ulaanbaatar [12], and increased coal consumption in winter is attributed to increased household use of coal-fired stoves. This increase in household coal consumption is partly due to population influx from rural areas to Ulaanbaatar, leading to the development of districts comprised of traditional tents with poor infrastructure (termed “ger” in Mongolia). People living in ger districts use smoke-rich fuel for household use, resulting in frequent smog and indoor air pollution in Ulaanbaatar. Indoor air pollution is a known important risk factor for COPD development [13–15], while other reports suggest that outdoor air pollution also affects lung function and respiratory symptoms although the effect seems to be small [1, 16–18].

The COPD prevalence in Ulaanbaatar is currently unknown. Here, we have conducted cross-sectional studies to assess the determinants and estimated prevalence of airflow limitation among Mongolian subjects aged 40–79 years living in Ulaanbaatar.

## Methods

### Study design

We conducted five cross-sectional studies among Mongolian adults aged 40 to 79 years at eight facilities (two hospitals and six community clinics) in Ulaanbaatar from 2012 to 2013 (May and September, 2012 and March, July, December, 2013). Participants were recruited by announcements advertising the health survey. Subjects received a self-completed questionnaire containing questions pertaining to age, gender, occupation, respiratory symptoms, and medical history. At the time of questionnaire administration, height and body weight were measured, and medical interviews, auscultations, and pulmonary function tests by respiratory specialists were conducted. Each survey included different sets of subjects and no duplicative subjects.

### Subjects

The present study included an initial 1,030 male and female community volunteers. Of these, 29 (2.8%) and 3 (0.3%) subjects were excluded due to ineligible age (< 40 or ≥ 80 years old) and lack of gender information, respectively. The remaining 998 (96.9%) were subjected to pulmonary function tests, however, the physician diagnosed that 71 (6.9%) subjects were contraindicated for spirometry due to conditions such as active tuberculosis, postpneumonectomy, or high blood pressure. Furthermore, spirometry on 24 (2.3%) subjects could not be performed due to a facility electric outage, and 57 (5.5%) subjects were excluded from analysis due to other reasons such as poor reproducibility, inappropriate spirograms, and/or mechanical issues. Lastly, 100 (9.7%) subjects were excluded due to restrictive ventilatory impairment (FEV1/FVC > 0.7 and FVC < 80% of predicted value). Characteristics of those subjects with restrictive ventilatory impairment was shown in S1 Table. Consequently, 746 (72.4%) subjects were eligible for further analysis.

### Pulmonary function test

A pre-bronchodilator pulmonary function test using the HI-105 spirometer (CHEST M.I., Inc., Tokyo, Japan) was conducted by the respiratory specialists. Spirometers were calibrated before each survey. At least three spirograms were obtained, and the highest forced expiratory volume in one second (FEV1) and forced ventilatory capacity (FVC) values were applied for diagnosis. Mongolian and Japanese respiratory specialists assessed the reproducibility and quality of spirograms (volume-time curve and flow-volume loop) and the invalid spirograms, which showed artefacts, insufficient inspiration, or blow-out, were excluded from analysis. Severity of airflow limitation was classified by the GOLD criteria (FEV1/FVC<0.7) with the predicted FEV1 defined by GLI2012 reference equations for North East Asians [1, 19].

### Data handling and statistical analyses

All data were anonymized, and managed as electronic data for the analysis. Prevalence of airflow limitation was age- and sex-standardized by direct standardization using Mongolian national population data published by the United Nations [19, 20]. Multiple logistic regression analyses were carried out by forced inclusion procedure for all the variables. Odds ratios (ORs) for presenting airflow limitation were adjusted for age group, sex (coded as 0 for female and 1 for male), body mass index (BMI) (coded as 0 for BMI ≥ 25.0 and 1 for BMI < 25.0), smoking status (coded as 0 for non-smokers, 1 for former smokers, and 2 for current smokers), household fuel (coded as 0 for smoke-free fuels such as gas and electricity and 1 for smoke-rich fuels such as coal, wood and dry animal dung fuel), and residential district (coded as 0 for urban areas and 1 for ger districts). The omnibus tests of model coefficient were shown to be significant (P < 0.01) for all models. Correlation coefficients (r) among predictive values were tested, and all r-values were less than 0.55, showing no multicollinearity. Statistical analyses including Welch's t-test for parametric analysis of two groups, χ2-test for analysis of categorical data, and multiple logistic regression analysis were performed using the statistical software package JMP version 11 (SAS Institute Inc., Cary, NC, USA). P values less than 0.05 on both sides were considered to be statistically significant.

### Ethics, consent, and permissions

The present study was approved by the Clinical Ethical Review Board of Kurume University School of Medicine. Before investigation, the participants were provided with explanations in person as to the purpose and method of the study, as well as information regarding the handling of the results. The study was carried out upon receipt of written consent.

## Results

### Characteristics of participants

Mongolian subjects (n = 1,030) were recruited from the general population in Ulaanbaatar. There were 746 (72.4%) eligible subjects for this study after excluding subjects who were out of the age range, unknown gender, invalid spirometry, in poor physical condition, or with restrictive ventilatory impairment. Characteristics of participants are presented in Table 1. Mean ages of males and females were similar. Male-to-female ratio was 0.53 (258: 488), and BMI, smoking status, residential district, and severity of airflow limitation according to GOLD criteria were significantly different between sexes. When subjects were compared with the general Mongolian population of the same age group in 2012 to 2013 [20, 21], the age characteristic of this study population was slightly older with a significantly lower percentage of male subjects (34.6% of the study subjects) than the general population (47.4% of the general population) (Table 2).

**Table 1:** Characteristics of the study subjects with valid spirometry.

Characteristics	Male		Female		Statistical analysis
**Age**					
(Mean ± SD)	54.1 ± 10.7		54.1 ± 9.5		N.S.
**BMI (kg/m**^**2**^**)**					
(Mean ± SD)	25.8 ± 4.0		27.9 ± 4.9		P < 0.001
	N	%	N	%	
**Obese and overweight (BMI≥25.0)**	128	49.6	347	71.1	P < 0.001
**Normal and underweight (BMI<25.0)**	130	50.4	141	28.9	
**Smoking status**					
Never smoker	88	34.1	416	85.2	P < 0.001
Former smoker	32	12.4	21	4.3	
Current smoker	138	53.5	51	10.5	
**Household fuel**					
Smoke-free	89	34.5	199	40.8	N.S.
Smoke-rich	169	65.5	289	59.2	
**Residential district**					
Urban area	100	38.8	256	52.5	P < 0.001
Ger district	158	61.2	232	47.5	
**Airflow limitation**					
Stage I	18	7.0	10	2.0	P < 0.005
Stage II	19	7.4	33	6.8	
Stage III + IV	4	1.6	2	0.4	
None	217	84.1	443	90.8	
**Total (n = 746)**	258	34.6	488	65.4	

**Table 2:** Prevalence of airflow limitation in study population standardized by Mongolian population.

Characteristic	Study subject		Mongolian population (40–79 years old)				Prevalence of airflow limitation (%)						
			Male		Female		Crude						Standardized
Age group	N	%	N (× 10^3^)	%	N (× 10^3^)	%	Male		Female		Overall		(by sex)
							N	%	N	%	N	%	%
40–49	267	35.8	178	50.6	186	47.5	6	6.3	8	4.7	14	5.2	5.5
50–59	264	35.4	106	30.1	121	31.0	15	17.2	15	8.5	30	11.4	12.6
60–69	146	19.6	46	13.1	54	13.8	12	27.9	12	11.7	24	16.4	19.1
70–79	69	9.2	22	6.3	30	7.7	8	25.0	10	27.0	18	26.1	26.2
Total	746	100.0	352	100.0	392	100.0							12.4
**Sex**													**(by age)**
Female	488	65.4	-	-	392	52.7	-	-	45	9.2	86	11.5	8.5
Male	258	34.6	352	47.3	-	-	41	15.9	-	-			13.6
							Age- and sex-standardized prevalence						10.9

### Expected prevalence of airflow limitation

Based on spirometry data, the unadjusted prevalence of airflow limitation in the study subjects was 11.5% (Table 2). Severity of airflow limitation according to the GOLD criteria using the GLI 2012 reference equations for North East Asians was: mild, 43.9% (n = 18, male) and 22.2% (n = 10, female); moderate, 46.3% (n = 19, male) and 73.3% (n = 33, female); and severe or higher, 9.8% (n = 4, male) and 4.4% (n = 2, female). No females were classified as having very severe airflow limitation. Crude and sex-standardized prevalence of airflow limitation were increased with increasing age, and crude and age-standardized prevalence of airflow limitation in males were higher than in females (Table 2). Age- and sex-standardized prevalence of airflow limitation was 10.9%. The prevalence of airflow limitation stratified by subgroup is shown in Table 3. Multiple logistic regression analyses to estimate odds ratios for presenting airflow limitation were performed using age, sex, BMI, smoking status, household fuel type, and residential district as predictive variables. Crude ORs were significant higher in older subjects, males, the lower BMI group (< 25), and in current smokers. Of these, an older age, a lower BMI, and current smoking status maintained significance after adjustment (Table 3). The prevalence for subjects living in ger districts was higher than those in urban area but OR was not significant.

**Table 3:** Factors affecting the prevalence of airflow limitation.

Characteristics	Prevalence (%)		Logistic regression			
	Crude	Standardized	Unadjusted		Adjusted	
Age group		(by sex)	OR	95% CI	OR	95% CI
40–49	5.2	5.5	1.00	(ref)	1.00	(ref)
50–59	11.4	12.6	2.32	1.22–4.60	2.53	1.32–5.09
60–69	16.4	19.1	3.56	1.80–7.28	4.06	2.02–8.45
70–79	26.1	26.2	6.38	3.00–13.86	8.66	3.89–19.81
		**(by age)**				
**Sex**	**Male (Female)**	**Male (Female)**				
Male	15.9 (9.2)	13.6 (8.5)	1.86	1.18–2.93	1.13	0.63–2.03
		**(by age and sex)**				
**BMI**	**< 25.0 (≥ 25.0)**	**< 25.0 (≥ 25.0)**				
< 25.0	16.6 (8.6)	14.8 (8.2)	2.11	1.34–3.32	2.05	1.27–3.32
**Smoking status**						
Never	9.7	8.5	1.00	(ref)	1.00	(ref)
Former	9.4	6.1	0.97	0.32–2.34	0.81	0.26–2.11
Current	16.9	14.8	1.89	1.16–3.05	1.91	1.02–3.56
**Household fuel**	**Smoke-rich (Smoke-free)**	**Smoke-rich (Smoke-free)**				
Smoke-rich	11.4 (11.8)	8.0 (9.9)	0.96	0.61–1.53	0.79	0.45–1.39
**Residential area**	**Ger district (Urban area)**	**Ger district (Urban area)**				
Ger district	13.1 (9.8)	12.4 (8.4)	1.38	0.88–2.19	1.55	0.88–2.77

### Differentiation of asthma from putative COPD

To assess the possibility of asthma, the study population was stratified by the number of affirmative responses to four asthma-related questions; self-reported history of asthma, history of wheezing in the past 12 months, frequent or occasional wheezes, and physician-diagnosed asthma (Table 4). The number of subjects with physician-diagnosed asthma was 17 (4 male; 13 female) (data not shown). There were 8 subjects (2 male; 6 female) with physician-diagnosed asthma also had airflow limitation. The crude prevalence of airflow limitation ranged from 4.3% to 11.5% depending on the number of affirmative responses to asthma-related questions. The prevalence ranged from 4.0% to 10.9% when they were standardized by age and sex. When subjects with physician-diagnosed asthma were excluded from putative COPD, the crude prevalence of putative COPD was 10.5% (age- and sex-adjusted prevalence: 10.0%) in the study population.

**Table 4:** Prevalence of putative COPD by diagnostic category of asthma.

Diagnosis criteria for asthma^a^	Possible asthma [n (%)]	Putative COPD [n (%)]	Prevalence of putative COPD in study population (%)	
			Crude	Age- and sex-standardized
None of four criteria affirmative	54 (62.8)	32 (37.2)	4.3	4.0
Any one of four criteria affirmative	36 (41.9)	50 (58.1)	6.7	6.2
Any two of four criteria affirmative	16 (18.6)	70 (81.4)	9.4	8.7
Any three of four criteria affirmative	4 (4.7)	82 (95.3)	11.0	10.4
Four of four criteria affirmative	0 (0.0)	86 (100.0)	11.5	10.9
Physician-diagnosed asthma	8 (9.3)	78 (90.7)	10.5	10.0

^a^ Criteria for asthma diagnosis: self-reported history of asthma; history of wheezing in the past 12 months; frequent or occasional wheezes; physician-diagnosed asthma

## Discussion

This is the first study on the prevalence of airflow limitation in Mongolia. We investigated lung function in Mongolian subjects aged 40 to 79 years living in Ulaanbaatar and found that the crude prevalence of airflow limitation was 11.5%. Multiple logistic regression analysis revealed that an older age, lower BMI, and current smoking status were independent predictive variables for airflow limitation. In this study, COPD may be confounded by the presence of asthma as post-bronchodilator spirometry was not conducted. When subjects with airflow limitation were stratified according to different criteria for asthma, putative COPD prevalence varied between 4.3 to 11.5%.

The prevalence of COPD shows interstudy variation depending on the diagnostic criteria. Halbert et al. reported that the pooled prevalence of 37 estimates for COPD was 7.6% (95% Confidence interval (CI): 6.0–9.5) [6]. Of these 37 estimates, the differences based on criteria were relatively large, for example, spirometric criteria indicated 9.2% prevalence while patient-reported diagnosis resulted in 4.9%. The most popular criteria using spirometric results were based on the GOLD criteria [1, 6]. The crude prevalence of airflow limitation (GOLD stage I (FEV1/FVC < 0.7)) in the present study was 11.5% when the possibility of asthma was not considered and 10.5% when subjects with physician-diagnosed asthma were excluded from putative COPD (Table 4). Furthermore, the age- and sex-standardized prevalence excluding subjects with physician-diagnosed asthma was 10.0%. When criterion used in BOLD study (GOLD stage II or higher (FEV1/FVC < 0.7 and FEV1 < 80% of predicted value)) was employed, the crude prevalence of airflow limitation was 7.8% and standardized prevalence was 7.3% (S2 Table) [6]. Age- and sex-standardized prevalence excluding physician-diagnosed asthma was 6.6% (S3 Table). Our results are comparable with other prevalence studies [6–9].

BMI was significantly lower in subjects with airflow limitation than those with normal lung function (28.1 ± 5.0 (Normal) vs. 25.7 ± 2.9 (Airflow limitation), P < 0.001). Due to the cross-sectional design of the present study, it is not clear whether the low BMI is a risk factor or secondary to airflow limitation, although low BMI has been reported to be a risk factor for COPD development [22, 23]. The present study also showed that lower BMI was one of the independent predictive variables for presenting airflow limitation, suggesting that low BMI is strongly associated with COPD development. Smoking and second-hand smoke have been reported to be the most important risk factors for COPD development [1, 24, 25]. Smoking rates in Asian countries are high especially for males [26], and in the present study (Table 1). Crude OR for airflow limitation was significantly higher in males (Table 3), but significance was lost when OR was adjusted, reflecting the high smoking prevalence in male subjects. A current smoking status was found to be a significant risk factor for airflow limitation even after the adjustment (Table 3). Smoking cessation or repeated attempts at smoking cessation has been reported to be effective in preventing lung function decline and decreasing the prevalence of respiratory symptoms even with subsequent cessation failure [27–31]. In contrast to current smokers, former smoking was not significant risk factor for airflow limitation although the number of former smokers in the present study was small (Table 3). Therefore, we can speculate that smoking cessation is effective in Mongolia for preventing lung function deterioration. Furthermore, for the subjects with airflow limitation of GOLD stage II or higher, both current and former smoking were not significant risk factors for airflow limitation (S4 Table). These results suggest that smoking does not have large impact on the risk for moderate to severe airflow limitation (GOLD stage II or higher) but for mild (GOLD stage I) airflow limitation in this population. It is consistent with the higher prevalence of mild airflow limitation (GOLD stage I) in male which showed higher smoking prevalence than in female. In general, people using smoke-rich fuel such as wood and coal for household use were exposed to indoor air pollution. Exhausted smoke from gers is a primary cause of ambient air pollution in ger districts. Thus, people living in ger districts are exposed to ambient air pollution for longer periods than people living in urban area although air pollution covers urban areas as well, depending on time and direction of the wind. Since indoor and outdoor air pollution is reported to be a cause of airflow limitation [1, 13–18], we included the residential district and household fuel type as independent variables in the multivariate analysis. Here, residential area and household fuel type was not a significant predictor of airflow limitation (Table 3). Since actual exposure to air pollution was not measured in the present study, subject responses accurately reflect exposure to air pollution cannot be validated. The prevalence of airflow limitation in ger districts was higher than that in urban areas but did not reach significance (Table 3). This result suggests that the effect of air pollution on airflow limitation is relatively small in the present study. Our previous study suggested that subjects living in ger districts with ventilatory impairment showed significantly higher prevalence of respiratory symptoms, and decreased health status in the cold season (unpublished data). Therefore, we can speculate that the effect of air pollution was one of the risk factor for the exacerbation of symptoms and health status rather than for the decline in lung function.

Since patients do not present themselves to the physician for examination until they have severe symptoms or significant impairment, COPD is likely underestimated [32]. We assessed the multivariate model for predicting airflow limitation using respiratory symptoms as independent variables to encourage subjects with certain symptoms to consult a physician for early detection of COPD. In this study, subjects presenting with the symptom “usually cough up sputum first thing in the morning” were liable to develop airflow limitation even after adjustment (S1 Fig). Our result is consistent with a report by Kessler et al., where the fluctuation of COPD symptoms over the day in COPD patients and all COPD-related symptoms including phlegm were most predominant upon waking in the morning [33]. Encouraging subjects who suffer respiratory symptoms in the morning to consult a physician would be effective for early diagnosis of COPD and reduction of its associated burdens.

There are several limitations in this study. First, whether the putative risk factors found in the present study indicates a risk of airflow limitation or its consequences is unclear due to the cross-sectional design of this study. Second, in the present study, postbronchodilator spirometry to distinguish asthma from COPD could not be performed as this study was conducted as a screening. Since the biggest concern about accuracy of the estimation in this study was how many subjects with pure asthma without COPD were included in the subjects with airflow limitation, we considered the possibility of bronchial asthma in Table 4 using differential diagnostic criteria. As shown in Table 4, the standardized prevalence ranged 4.0% to 10.9% corresponding the proportion of false positive ranged 63.3% to 0%. In Mongolia, asthma prevalence was reported to be 4.7% based on symptoms [34], and Viinanen et al. reported the prevalence of asthma in Ulaanbaatar to be 2.1% based on spirometry data although both studies did not show COPD prevalence [35]. It is difficult to distinguish asthma from COPD in smokers and older adults [36], as some patients may have clinical features of both asthma and COPD known as asthma-COPD overlap syndrome (ACOS). In the present study, 2.3% of subjects were diagnosed with asthma by respiratory specialists based on the symptoms and medical history (Table 4). This is comparable to asthma prevalence in Ulaanbaatar based on spirometry data [35]. Lastly, the age distribution and male-to-female ratio of subjects were biased in the present study as study subjects were older and with a lower male-to-female ratio compared to the general Mongolian population. The overall prevalence of airflow limitation in this study might be affected by the lower male-to-female ratio because the proportion of smokers was much higher in males than in females. Nonetheless, this study population covered approximately 0.1% of the Mongolian population aged 40 to 79 years [20, 21] and the direct standardization by age and sex were carried out using population data [20]. Furthermore, multivariate analyses adjusted for age, sex, and other factors potentially affecting airflow limitation were carried out. Hence, the results obtained in this study reflect the actual situation in Mongolia although generalizations are limited.

## Conclusions

We investigated the prevalence of airflow limitation in subjects aged 40 to 79 in Ulaanbaatar, Mongolia. The age- and sex-standardized prevalence of putative COPD ranged from 4.0 to 10.9% depending on the asthma criteria and the age- and sex-standardized prevalence was 10.0% when patients with physician-diagnosed asthma were excluded from COPD. An older age, lower BMI, and current smoking status were independent risk factors for airflow limitation. The putative COPD prevalence in this study was consistent with other reports from Asian countries.

## Supporting information

S1 Fig Respiratory symptoms associated with airflow limitation. Multiple logistic regression analyses were carried out by a stepwise selection procedure used with forced inclusion of specific variables. Respiratory symptoms were added as additional predictive variables as follows; 1. Does the weather affect your cough? (Yes/No or No cough); 2. Have you ever coughed up sputum from your chest when you do not have a cold? (Yes/No); 3. Do you usually cough up sputum from your chest first thing in the morning? (Yes/No); 4. How frequently do you wheeze? (Occasionally or more often/Never); 5. Do you have or have you had any allergies? (Yes/No); 6. Do you suffer from any infectious disease? (Yes/No). Odds ratios were adjusted for age group, sex, BMI, smoking status, household fuel, and residential district. The vertical short line represents odds ratios; the horizontal line represents 95% confidence interval. The omnibus tests of model coefficient were shown to be significant (P < 0.01) for all models. Correlation coefficients (r) among predictive values were tested, and all r-values were less than 0.55, indicating no multicollinearity. P values less than 0.05 were considered to be statistically significant.
(TIF)

S1 Table Characteristics of the subjects with restrictive ventilatory impairment. (DOCX)

S2 Table Prevalence of airflow limitation in study population standardized by Mongolian population when the cutoff value of airflow limitation was set at FEV1/FVC < 0.7 and FEV1 < 80% of predicted value (GOLD stage II or higher). (DOCX)

S3 Table Prevalence of putative COPD (GOLD stage II or higher) by diagnostic category of asthma. (DOCX)

S4 Table Factors affecting the prevalence of airflow limitation (GOLD stage II or higher). (DOCX)
