# Supplementary figures and images for: Prevalence and risk factors of airflow limitation in a Mongolian population in Ulaanbaatar: Cross-sectional studies
Source: PLoS One. 2017 Apr 11;12(4):e0175557. doi: 10.1371/journal.pone.0175557 (PMC5388497; doi:10.1371/journal.pone.0175557)

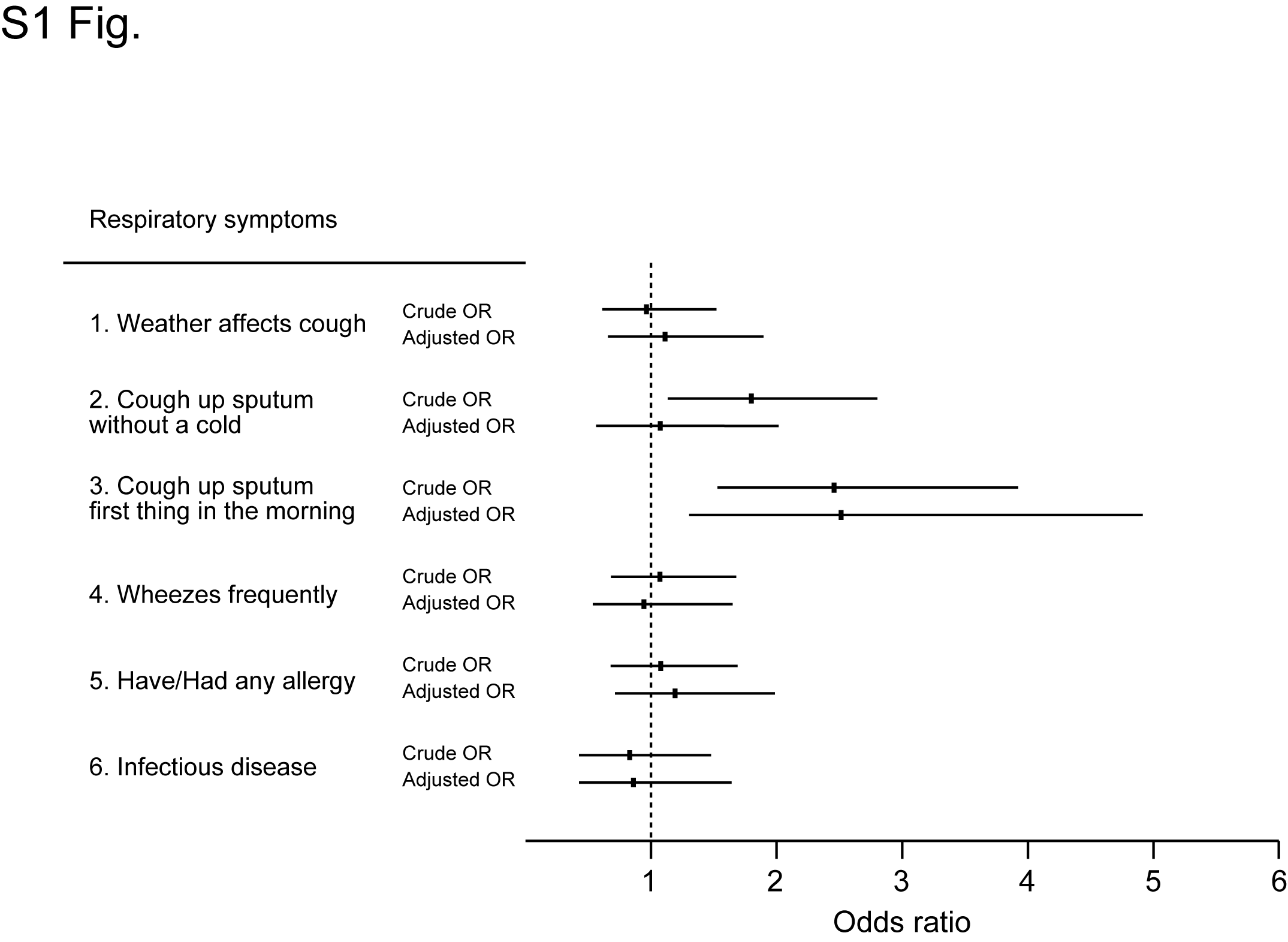

Supplement: S1 Fig — Multiple logistic regression analyses were carried out by a stepwise selection procedure used with forced inclusion of specific variables. Respiratory symptoms were added as additional predictive variables as follows; 1. Does the weather affect your cough? (Yes/No or No cough); 2. Have you ever coughed up sputum from your chest when you do not have a cold? (Yes/No); 3. Do you usually cough up sputum from your chest first thing in the morning? (Yes/No); 4. How frequently do you wheeze? (Occasionally or more often/Never); 5. Do you have or have you had any allergies? (Yes/No); 6. Do you suffer from any infectious disease? (Yes/No). Odds ratios were adjusted for age group, sex, BMI, smoking status, household fuel, and residential district. The vertical short line represents odds ratios; the horizontal line represents 95% confidence interval. The omnibus tests of model coefficient were shown to be significant (P < 0.01) for all models. Correlation coefficients (r) among predictive values were tested, and all r-values were less than 0.55, indicating no multicollinearity. P values less than 0.05 were considered to be statistically significant. (TIF) [file pone.0175557.s001.tif]
