# Supplementary material for: Prevalence and risk factors of airflow limitation in a Mongolian population in Ulaanbaatar: Cross-sectional studies
Source: PLoS One. 2017 Apr 11;12(4):e0175557. doi: 10.1371/journal.pone.0175557 (PMC5388497; doi:10.1371/journal.pone.0175557)
Supplement: S1 Table — (DOCX) [file pone.0175557.s002.docx]

**S1 Table. Characteristics of the subjects with restrictive ventilatory impairment.**

| **Characteristics** | **Male** | | **Female** | | **Statistical analysis** |
| --- | --- | --- | --- | --- | --- |
| **Age** |  |  |  |  |  |
| (Mean ± SD) | 56.4 ± 10.7 | | 57.9 ± 9.2 | | N.S. |
| **BMI (kg/m^2^)** |  | |  | |  |
| (Mean ± SD) | 26.7 ± 6.3 | | 28.8 ± 5.2 | | N.S. |
|  | N | % | N | % |  |
| **Obese and overweight (BMI≥25.0)** | 17 | 51.5 | 53 | 79.1 | P < 0.01 |
| **Normal and underweight (BMI<25.0)** | 16 | 48.5 | 14 | 20.9 |  |
| **Smoking status** |  |  |  |  |  |
| Never smoker | 9 | 27.3 | 57 | 85.1 | P < 0.001 |
| Former smoker | 6 | 18.2 | 2 | 3.0 |  |
| Current smoker | 18 | 54.5 | 8 | 11.9 |  |
| **Household fuel** |  |  |  |  |  |
| Smoke-free | 9 | 27.3 | 29 | 45.3 | N.S. |
| Smoke-rich | 24 | 72.7 | 35 | 54.7 |  |
| **Residential district** |  |  |  |  |  |
| Urban area | 18 | 54.5 | 42 | 62.7 | N.S. |
| Ger district | 15 | 45.5 | 25 | 37.3 |  |
| **Airflow limitation** |  |  |  |  |  |
| Stage I | 0 | 0.0 | 0 | 0.0 | N.S. |
| Stage II | 6 | 18.2 | 8 | 11.9 |  |
| Stage III + IV | 3 | 9.1 | 6 | 9.0 |  |
| None | 24 | 72.7 | 53 | 79.1 |  |
| **Total (n = 100)** | 33 | 33.0 | 67 | 65.4 |  |
