# Supplementary material for: Prevalence and risk factors of airflow limitation in a Mongolian population in Ulaanbaatar: Cross-sectional studies
Source: PLoS One. 2017 Apr 11;12(4):e0175557. doi: 10.1371/journal.pone.0175557 (PMC5388497; doi:10.1371/journal.pone.0175557)
Supplement: S2 Table — (DOCX) [file pone.0175557.s003.docx]

**S2 Table. Prevalence of airflow limitation in study population standardized by Mongolian population when the cutoff value of airflow limitation was set at FEV1/FVC < 0.7 and FEV1 < 80% of predicted value (GOLD stage II or higher).**

| **Characteristic** | **Study subject** | | **Mongolian population**  **(40 – 79 years old)** | | | | | **Prevalence of airflow limitation with FEV_1_ < 80% of predicted value** | | | | | | | | | |  |
| --- | --- | --- | --- | --- | --- | --- | --- | --- | --- | --- | --- | --- | --- | --- | --- | --- | --- | --- |
|  |  |  |  |  |  |  |  | **Crude** | | | | | | | | **Standardized** |  |  |
| **Age group** |  |  | **Male** | | **Female** | | | **Male** | | | **Female** | | | **Overall** | | **(by sex)** | |  |
|  | **N** | **%** | **N**  **(×10^3^)** | **%** | **N**  **(×10^3^)** | **%** | **N** | | **%** | **N** | | **%** | **N** | | **%** | **%** | | |
| 40 – 49 | 267 | 35.8 | 178 | 50.6 | 186 | 47.5 | 3 | | 6.3 | 7 | | 4.1 | 10 | | 3.8 | 3.6 | | |
| 50 – 59 | 264 | 35.4 | 106 | 30.1 | 121 | 31.0 | 13 | | 17.2 | 15 | | 8.5 | 28 | | 10.6 | 11.5 | | |
| 60 – 69 | 146 | 19.6 | 46 | 13.1 | 54 | 13.8 | 5 | | 27.9 | 8 | | 7.8 | 13 | | 8.9 | 9.5 | | |
| 70 – 79 | 69 | 9.2 | 22 | 6.3 | 30 | 7.7 | 2 | | 25.0 | 5 | | 13.5 | 7 | | 10.1 | 10.4 | | |
| Total | 746 | 100.0 | 352 | 100.0 | 392 | 100.0 |  | |  |  | |  |  | |  | 8.0 | | |
| **Sex** |  |  |  |  |  |  |  | |  |  | |  |  | |  | **(by age)** | | |
| Female | 488 | 65.4 | - | - | 392 | 52.7 | - | | - | 35 | | 7.2 | 58 | | 7.8 | 6.7 | | |
| Male | 258 | 34.6 | 352 | 47.3 | - | - | 23 | | 8.9 | - | | - |  |  |  | 8.0 | | |
|  |  |  |  |  |  |  |  | | Age- and sex-standardized prevalence | | | | | | | 7.3 | |  |
