# Supplementary material for: Prevalence and risk factors of airflow limitation in a Mongolian population in Ulaanbaatar: Cross-sectional studies
Source: PLoS One. 2017 Apr 11;12(4):e0175557. doi: 10.1371/journal.pone.0175557 (PMC5388497; doi:10.1371/journal.pone.0175557)
Supplement: S3 Table — (DOCX) [file pone.0175557.s004.docx]

**S3 Table. Prevalence of putative COPD (GOLD stage II or higher) by diagnostic category of asthma.**

| Diagnosis criteria for asthma^a^ | Possible asthma  [n (%)] | Putative COPD  (GOLD stage II or higher) [n (%)] | Prevalence of putative COPD (GOLD stage II or higher) in study population (%) | |
| --- | --- | --- | --- | --- |
|  |  |  | Crude | Age- and sex-standardized |
| None of four criteria affirmative | 42 (72.4) | 16 (27.6) | 2.1 | 2.1 |
| Any one of four criteria affirmative | 27 (46.6) | 31 (53.4) | 4.2 | 3.9 |
| Any two of four criteria affirmative | 12 (20.7) | 46 (79.3) | 6.2 | 5.6 |
| Any three of four criteria affirmative | 3 (5.2) | 55 (94.8) | 7.4 | 6.9 |
| Four of four criteria affirmative | 0 (0.0) | 58 (100.0) | 7.8 | 7.3 |
| Physician-diagnosed asthma | 6 (10.3) | 52 (89.7) | 7.0 | 6.6 |

^a^ Criteria for asthma diagnosis: self-reported history of asthma; history of wheezing in the past 12 months; frequent or occasional wheezes; physician-diagnosed asthma.
