# Supplementary material for: Prevalence and risk factors of airflow limitation in a Mongolian population in Ulaanbaatar: Cross-sectional studies
Source: PLoS One. 2017 Apr 11;12(4):e0175557. doi: 10.1371/journal.pone.0175557 (PMC5388497; doi:10.1371/journal.pone.0175557)
Supplement: S4 Table — (DOCX) [file pone.0175557.s005.docx]

**Table 4. Factors affecting the prevalence of airway obstruction with FEV_1_ predicted <80%.**

| **Characteristics** | **Prevalence (%)** | | **Logistic regression** | | | |
| --- | --- | --- | --- | --- | --- | --- |
|  | **Crude** | **Standardized** | **Unadjusted** | | **Adjusted** | |
| **Age group** |  | **(by sex)** | **OR** | **95% CI** | **OR** | **95% CI** |
| 40 – 49 | 3.7 | 3.6 | 1.00 | (ref) | 1.00 | (ref) |
| 50 – 59 | 10.6 | 10.6 | 3.05 | 1.50 – 6.72 | 3.19 | 1.56 – 7.05 |
| 60 – 69 | 8.9 | 9.5 | 2.51 | 1.08 – 6.03 | 2.64 | 1.13 – 6.38 |
| 70 – 79 | 10.1 | 10.4 | 2.90 | 1.02 – 7.86 | 3.25 | 1.11 – 9.11 |
|  |  | **(by age)** |  |  |  |  |
| **Sex** | **Male (Female)** | **Male (Female)** |  |  |  |  |
| Male | 8.9 (7.2) | 6.7 (8.0) | 1.27 | 0.72 – 2.18 | 1.05 | 0.51 – 2.08 |
|  |  | **(by age and sex)** |  |  |  |  |
| **BMI** | **< 25.0 (≥ 25.0)** | **< 25.0 (≥ 25.0)** |  |  |  |  |
| < 25.0 | 10.4 (6.3) | 9.6 (6.4) | 1.72 | 1.00 – 2.95 | 1.74 | 0.99 – 3.06 |
| **Smoking status** |  |  |  |  |  |  |
| Never | 7.5 | 6.7 | 1.00 | (ref) | 1.00 | (ref) |
| Former | 5.7 | 5.3 | 0.74 | 0.17 – 2.13 | 0.66 | 0.15 – 2.04 |
| Current | 9.0 | 6.6 | 1.21 | 0.65 – 2.17 | 1.12 | 0.52 – 2.34 |
| **Household fuel** | **Smoke-rich (Smoke-free)** | **Smoke-rich (Smoke-free)** |  |  |  |  |
| Smoke-rich | 7.9 (7.6) | 7.4 (6.6) | 1.03 | 0.60 – 1.82 | 0.88 | 0.46 – 1.69 |
| **Residential area** | **Ger district (Urban area)** | **Ger district (Urban area)** |  |  |  |  |
| Ger district | 8.7 (6.7) | 8.2 (5.9) | 1.32 | 0.77 – 2.30 | 1.38 | 0.72 – 2.66 |
